# Advancing herbal medicine: enhancing product quality and safety through robust quality control practices

**DOI:** 10.3389/fphar.2023.1265178

**Published:** 2023-09-25

**Authors:** Hongting Wang, Ying Chen, Lei Wang, Qinghui Liu, Siyu Yang, Cunqin Wang

**Affiliations:** Anhui Provincial Engineering Laboratory for Screening and Re-evaluation of Active Compounds of Herbal Medicines in Southern Anhui, Anhui Provincial Engineering Research Center for Polysaccharide Drugs, Anhui Innovative Center for Drug Basic Research of Metabolic Diseases, School of Pharmacy, Wannan Medical College, Wuhu, China

**Keywords:** herbal medication products, quality control, standardization, quality assurance protocols, quality control methods

## Abstract

This manuscript provides an in-depth review of the significance of quality control in herbal medication products, focusing on its role in maintaining efficiency and safety. With a historical foundation in traditional medicine systems, herbal remedies have gained widespread popularity as natural alternatives to conventional treatments. However, the increasing demand for these products necessitates stringent quality control measures to ensure consistency and safety. This comprehensive review explores the importance of quality control methods in monitoring various aspects of herbal product development, manufacturing, and distribution. Emphasizing the need for standardized processes, the manuscript delves into the detection and prevention of contaminants, the authentication of herbal ingredients, and the adherence to regulatory standards. Additionally, it highlights the integration of traditional knowledge and modern scientific approaches in achieving optimal quality control outcomes. By emphasizing the role of quality control in herbal medicine, this manuscript contributes to promoting consumer trust, safeguarding public health, and fostering the responsible use of herbal medication products.

## 1 Introduction

Herbal medication products have been utilized for centuries as a primary form of healthcare in many cultures worldwide (Motti, 2021). Derived from plants and plant-derived materials, these products offer a rich source of bioactive compounds with potential therapeutic benefits (Chrysant and Chrysant, 2017). The use of herbal medicines spans diverse traditional systems, such as traditional Chinese medicine (TCM), ayurveda, and indigenous healing practices (Liu et al., 2012; Kabak and Dobson, 2017). One of the key reasons for the enduring popularity of herbal medication products is their perceived natural origin and historical use. Many societies have a long-established tradition of utilizing herbal remedies to address a wide range of ailments and promote overall wellbeing (Liu et al., 2006; Alipour et al., 2022). This traditional knowledge has been passed down through generations, offering valuable insights into the healing properties of various plant species (Cho and Kim, 2020). Herbal medication products have demonstrated therapeutic potential across various health conditions, including digestive disorders, respiratory ailments, chronic pain, and immune system support (Ernst, 1998; Kaefer and Milner, 2008; Shaikh et al., 2020). Their bioactive constituents, such as alkaloids, flavonoids, terpenes, and polyphenols, can interact with biological systems, offering potential therapeutic benefits (Wu et al., 2005; Wang and Liu, 2014; Brown, 2017). However, the growing demand for herbal medication products also raises concerns about quality control, safety, and efficacy (Osman et al., 2019; Zhou et al., 2019; Tao et al., 2023). Ensuring the consistent quality and standardization of herbal products is crucial to guarantee their safety, efficacy, and reproducibility (Rahath Kubra et al., 2016). Rigorous quality control measures are essential to protect the health and safety of consumers (Deutch et al., 2019). By implementing stringent testing and quality assurance protocols, potential risks such as contamination, adulteration, and the presence of harmful substances can be minimized, ensuring that the herbal medication products are safe for consumption (Giacometti et al., 2018). Insufficient quality control can pose serious safety risks to consumers. Contaminants, adulterants, or incorrect formulations in herbal medication products may lead to adverse reactions, toxicity, or other health complications (Chen et al., 2018; Cai et al., 2019). Inadequate quality control may result in inconsistent levels of active compounds in herbal medication products (Liu et al., 2020). This inconsistency can lead to variable therapeutic effects, making it challenging for healthcare professionals to prescribe and manage patient treatments effectively (Ma et al., 2022). Quality control measures help maintain consistency in the composition and potency of herbal medication products (Yago and Pla, 2020). Through standardized manufacturing processes and testing methods, the levels of active compounds can be monitored and controlled, ensuring that the products deliver the desired therapeutic effects consistently (Parr et al., 2001; Braga et al., 2020). This commitment helps build trust among consumers, healthcare professionals, and regulatory authorities. The purpose of the review is to provide a comprehensive overview of the importance, methods, and considerations involved in ensuring the quality, efficiency, and safety of herbal medication products.

## 2 Overview of herbal medication products

**FIGURE1:**
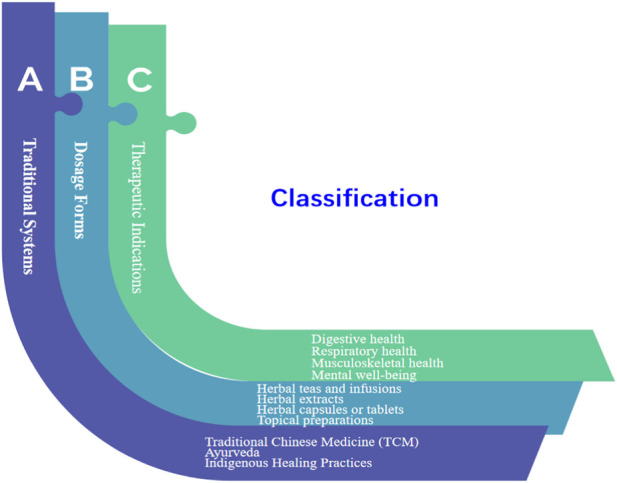
A classification of herbal medication products.

Herbal medication products, also known as herbal medicines or phytotherapeutic products, refer to medicinal products derived from plants or plant materials (Liu et al., 2013). These products utilize the therapeutic properties of various plant species, including their leaves, flowers, roots, stems, or extracts, to promote health and treat or prevent diseases (Sinha et al., 2015). Herbal medication products often contain a combination of active compounds, such as alkaloids, flavonoids, terpenes, and phenolic compounds, which contribute to their pharmacological effects (Chang, 2000; Ranasinghe et al., 2015). Herbal medication products can be classified based on different criteria. Based on different classification methods, we summarize the classification rules of traditional Chinese herbal medicine (Figure 1). Traditional Chinese medicine (TCM) and Ayurvedic herbal medication products have both holistic system of healthcare and healing that has been practiced for over 2,000 years in China and other parts of East Asia (Balachandran and Govindarajan, 2005; Joshi et al., 2017; Prasad et al., 2021). TCM uses a wide range of medicinal herbs, minerals, and animal products to restore balance and treat various health conditions (Wang et al., 2022). These natural substances have been used for centuries for their therapeutic properties and health benefits (Dina et al., 2022). Herbal formulas are often prescribed based on the individual’s unique pattern of disharmony (Zhu et al., 2019). Different herbs have specific chemical compounds that exert various effects on the body. For example, echinacea is used to boost the immune system, ginger for digestive issues, and ginkgo biloba for cognitive function (Karsch-Völk et al., 2014; Sharifi-Rad et al., 2018). Certain minerals and mineral-rich substances are used in traditional medicine for their therapeutic effects. For instance, calcium, magnesium, iron, sulphur, and zinc were supplied for bone health and muscle function, the treatment of anemia and to boost hemoglobin levels, skin conditions like acne and eczema, the immune system, and wound healing (Harper et al., 1987; Song et al., 2021; Xiao et al., 2022). Traditional medicine systems, particularly in East Asia, have used animal products for their medicinal properties (Cheng et al., 2022). Some examples include deer antler velvet which is used to strengthen the body, improve energy, and support joint health, and bear bile used in some traditional Chinese remedies, though the use of bear bile is controversial due to animal cruelty concerns (Wu et al., 2013; Yu et al., 2017). Cordyceps is a fungus that parasitizes insects and is used for various health benefits, including respiratory support and energy enhancement (Chen et al., 2017; Lou et al., 2019; Ashraf et al., 2020; Yang et al., 2020). It is essential to note that while traditional medicine systems have been using these substances for generations, the safety and efficacy of medicinal herbs, minerals, and animal products are not always supported by modern scientific evidence (Yuan et al., 2016). Some of these substances may interact with medications or have potential side effects (Saini et al., 2022). Therefore, it is crucial to consult with qualified healthcare professionals, such as herbalists or traditional medicine practitioners, who have knowledge and experience in the safe use of these natural remedies. In modern times, there is an increasing interest in studying traditional medicinal practices and evaluating the therapeutic potential of these natural substances through rigorous scientific research (Schwabl and Vennos, 2015; Jaiswal et al., 2016). It is precisely because of the diversity of Chinese herbal medicines that the quality control of the herbal medicine industry is particularly important for the efficiency and safety of herbal medication products (Kankanamalage et al., 2014). So, integrative medicine approaches seek to combine the best practices from traditional and modern medicine to provide comprehensive and personalized healthcare solutions.

## 3 Quality control for herbal medication products

Quality control is a systematic approach that involves monitoring and controlling various aspects of herbal product development, manufacturing, and distribution to guarantee consistent product quality (Ding et al., 2008). Quality control measures are essential in any industry or organization as they play a significant role in ensuring that products or services meet the expected standards and specifications (Osman et al., 2019; Chen et al., 2023). Effective quality control measures involve evaluating processes and identifying areas for improvement (Qin et al., 2012; Zhao et al., 2018; Gong et al., 2023). By streamlining operations, eliminating bottlenecks, and addressing inefficiencies, organizations can enhance productivity and achieve higher output levels with fewer resources (Gong et al., 2023).

### 3.1 Standardization and identification of herbs

The first step is to built standardization and identification of herbs for effective quality control measures. Standardization and identification of herbs involve establishing consistent and reliable levels of active compounds or markers in herbal medication products (Aleksieva and Yordanov, 2018). It aims to minimize batch-to-batch variability and ensure that each product meets predetermined quality standards (Ketai et al., 2000). Identify the key active compounds or markers in the herb that contribute to its therapeutic properties. Some key aspects of them involve active compound identification, quantitative analysis, and reference standards, which can be done through scientific research, traditional knowledge, or existing literature (Zhao et al., 2022). Develop methods to quantitatively measure the levels of active compounds or markers (An et al., 2022). This can involve techniques such as chromatography (HPLC, GC), spectroscopy (UV-Vis, IR), or specific chemical assays (Pan et al., 2018). Establish reference standards or reference materials that represent the desired levels of active compounds or markers (Xiong et al., 2022). These standards act as benchmarks for comparison during quality control testing and help ensure consistency across batches (Jin et al., 2018). Standardization provides a means to monitor and control the quality and efficacy of herbal medication products, enabling healthcare professionals to prescribe treatments with confidence.

**FIGURE2:**
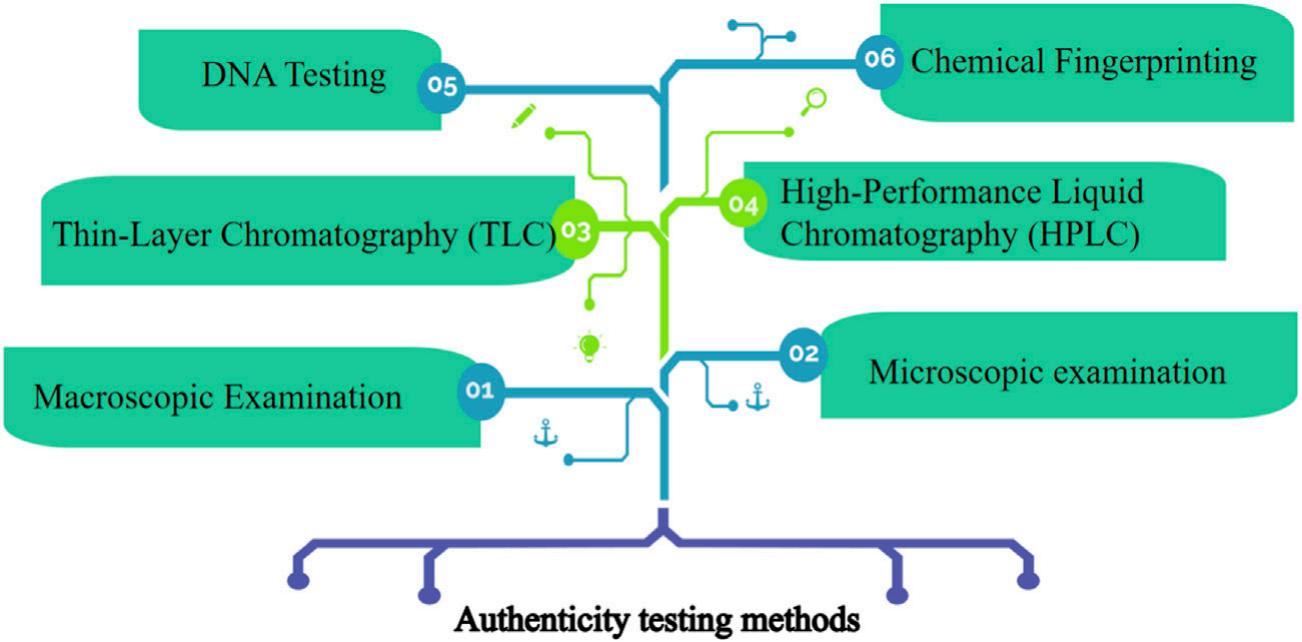
The quality control methods for the accurate identification and verification of the herbs.

Authenticity testing methods are essential in quality control for herbal medication products to ensure the accurate identification and verification of the herbs used to ensure that the correct herb is being used, as different species or plant parts may have varying therapeutic properties and safety profiles (Zhang et al., 2022). Herb identification is the process of accurately identifying the botanical species or plant material used in herbal medication products (Lo and Shaw, 2019). Some commonly employed authenticity testing methods involve macroscopic examination, microscopic examination, thin-layer chromatography, high-performance liquid chromatography, DNA Barcoding, and chemical profiling (Figure 2).

Macroscopic Examination involves visual inspection and examination of the physical characteristics of the herb, including its color, shape, size, texture, and any unique features (K et al., 2021). Macroscopic examination helps in identifying the herb and distinguishing it from other similar-looking plants. Microscopic examination involves the use of a microscope to examine the cellular structures and characteristic features of the herb (Xiong et al., 2019). This method helps in identifying specific plant parts, such as leaves, stems, or roots, and verifying their authenticity. TLC is a technique used to separate and analyze the chemical constituents of a herb. It involves applying a thin layer of the herb extract onto a solid support, which is then developed using a suitable solvent system (Poole, 2003; Cheng et al., 2011). The resulting chromatogram can be compared to reference standards to identify the herb and detect any adulterants or contaminants (Del Bubba et al., 2013). HPLC is a powerful analytical technique that separates and quantifies the chemical compounds in a herb (Sontag et al., 2019). It can be used to determine the presence and concentration of specific marker compounds or active ingredients, ensuring the consistency and quality of herbal medication products (Malherbe et al., 2012). DNA testing, specifically DNA barcoding, is a molecular technique used to authenticate and identify herbal species (Li et al., 2015). It involves sequencing a specific region of the herb’s DNA, such as the barcode region, which is unique to each species. By comparing the obtained DNA sequence with a reference database, the herb’s genetic identity can be determined, ensuring the use of the correct species and detecting any potential adulteration or substitution (DeSalle et al., 2005; Senapati et al., 2022). DNA testing provides a highly reliable method for herb identification and is particularly useful when the herbs are in processed or powdered forms. Next, chemical fingerprinting involves analyzing the chemical constituents of herbs using analytical techniques such as high-performance liquid chromatography (HPLC), gas chromatography-mass spectrometry (GC-MS), or nuclear magnetic resonance (NMR) (Wibowo et al., 2015; Li et al., 2022). By comparing the chemical profiles obtained from a herb sample with established reference profiles, manufacturers can verify the authenticity, consistency, and quality of the herbs. Chemical fingerprinting helps identify specific marker compounds or active ingredients, ensuring the desired potency and therapeutic efficacy of herbal medication products (Gainza et al., 2020). Chemical fingerprinting and DNA testing provide objective and scientific data for quality control in herbal medication products (Yin et al., 2022). By integrating these techniques into quality control processes, manufacturers can establish robust standards, maintain consistency, meet regulatory requirements, verify the authenticity of herbal ingredients, detect any adulterations or contaminants, and ensure the consistency and safety of herbal medication products. It is worth noting that macroscopic and microscopic examination should be complemented with other testing methods, such as Thin-Layer Chromatography (TLC), High-Performance Liquid Chromatography (HPLC), DNA barcoding, or chemical profiling, to provide a comprehensive quality control approach for herbal medication products (Chen et al., 2006; Hu et al., 2021). These authenticity testing methods help ensure the accurate identification and quality control of herbs used in herbal medication products that may compromise the safety and efficacy of the products.

### 3.2 Processing and manufacturing controls

Extraction methods play a crucial role in obtaining the desired active compounds from herbs during the manufacturing process of herbal medication products (Boutron et al., 2007; Abeillé et al., 2020). Optimizing extraction methods is critical to ensure the efficient extraction of bioactive compounds from herbs, which directly impacts the potency and efficacy of herbal medication products (Zhu et al., 2006). Some key points regarding extraction methods and optimization involve selection of solvent, pre-processing of herbal material, ratio of solvent to herbal material, temperature and time, extraction techniques, multiple extractions, pH adjustment, use of co-solvents or enhancers, quality control, safety considerations, sustainability, validation and reproducibility (Hawthorne et al., 2000; Boateng et al., 2023). The selection of the appropriate extraction technique depends on factors such as the nature of the herb, targeted bioactive compounds, desired product characteristics, and manufacturing scale (Gollahon and Holt, 2000). Each technique has its advantages and limitations, and it is important to choose the most suitable method for efficient extraction (Silva et al., 2022). First of all, the choice of solvent is crucial for successful extraction (Brennan et al., 2009). Different solvents have varying polarities and can selectively extract different compounds (Kalkan et al., 2019). Common solvents used include water, ethanol, methanol, and their mixtures. The solvent selection should consider the solubility of target compounds, potential toxicity, environmental impact, and regulatory requirements (Tasfiyati et al., 2022). It is important to optimize the solvent-to-herb ratio to achieve optimal extraction efficiency. Besides, optimization of extraction parameters such as temperature, time, and pressure is essential to maximize the extraction of bioactive compounds while maintaining their stability and minimizing degradation (Ma et al., 2021). These parameters can vary depending on the herb and its targeted compounds. Factors like temperature and extraction duration should be carefully controlled to prevent the loss of heat-sensitive compounds or the degradation of thermally unstable components (Feldmann and Bondemark, 2006). Apparently, pre-treatment techniques, such as size reduction (grinding, milling), drying, or specific treatments like blanching, can influence the efficiency of extraction (Wibowo et al., 2015; An et al., 2022). These techniques help in breaking down the plant matrix, facilitating the release of bioactive compounds and improving their accessibility to the extraction solvent (Dai et al., 2022). Once the extraction method is established, process validation should be performed to ensure consistent and reproducible extraction results. Process validation involves confirming that the extraction process consistently meets predetermined quality standards, including extraction efficiency, yield, and the presence of desired bioactive compounds (González et al., 2021; Kapadia et al., 2022). Validation parameters may include analytical testing, comparison with reference samples, and statistical analysis (Campuzano and González-Martínez, 2017; Onishi et al., 2018). Various extraction techniques and extraction parameters are listed, including maceration, percolation, reflux, Soxhlet extraction, ultrasound-assisted extraction, and supercritical fluid extraction (Table 1).

**TABLE 1:** Extraction methods and optimization factors.

Advanced extractions techniques	Ultrasound-assisted extraction (UAE)	Microwave-assisted extraction (MAE)	Pressurized liquid extraction	Supercritical fluid extraction	References
Sample	√	√	√	-	Wolle and Conklin (2018); Pradhan et al. (2019); Woolman and Liu (2022)
Extraction solvent	√	√	√	-	Rodsamran and Sothornvit (2019); Gil-Díaz et al. (2021)
Pressure	-	-	√	√	Chan and Choo (2013); Ma et al. (2017); Silva et al. (2022)
Extraction temperature	√	√	√	-	Brennan et al. (2009); Elfgen et al. (2020); Gil-Díaz et al. (2021)
Size	-	-	√	-	El Maangar et al. (2020); da Cunha et al. (2021); Ma et al. (2021)
PH	-	-	√	-	Feldmann and Bondemark (2006); Gupta and Sinha (2007); Spineli (2017); Tasfiyati et al. (2022)
Flow rate	-	-	√	-	Du et al. (2009); Backes et al. (2018); da Cunha et al. (2021)
Extraction time	√	√	√	√	Xie and Dreisinger (2009); González et al. (2021); Tsiaka et al. (2023)
Ultrasound power	√	-	-	-	Mulinacci et al. (2011); Boyd et al. (2012); Speltini et al. (2021)
Frequency	√	-	-	-	Hawthorne et al. (2000); Zhu et al. (2006); Silva et al. (2022)
Intensity	√	-	-	-	Backes et al. (2018); Speltini et al. (2021); Yin et al. (2022)
Microwave power	-	√	-	-	Byrd et al. (1991); Gollahon and Holt (2000); Ege and Demirkol (2021)
Plant matrix characteristic	-	√	-	-	Pihlström et al. (2002); El Maangar et al. (2020); González et al. (2021)

### 3.3 Quality assessment during processing

Quality assessment during processing is a crucial aspect of ensuring the safety, efficacy, and consistency of herbal medication products (Fillingim et al., 2016). It involves monitoring and evaluating various parameters and factors throughout the manufacturing process (Sargeant et al., 2022). Here, some key points regarding quality assessment were summarized during processing (Table 2).

**TABLE 2:** Quality management practices.

Quality assessment	Parameters	Methods	References
Raw Material Evaluation	Botanical Identity	Visual inspections Macroscopic examinations Microscopic examinations Chemical analyses	Stringham et al. (2009); Sridharan et al. (2016); Benedetti et al. (2019); Liang et al. (2023)
	Purity and Authenticity		
	Plant Part		
	Geographic Origin		
	Moisture Content		
	Foreign Matter		
	Chemical Composition		
	Microbial Contamination		
	Heavy Metal Content		
	Residual Solvents		
Good Manufacturing Practices (GMP)	Proper documentation Personnel training Hygiene practices Quality control procedures	Facility Design and Maintenance	Velagaleti et al. (2002); Burcham et al. (2018); Crimi et al. (2021); Lai et al. (2022)
		Personnel Training and Qualification	
		Standard Operating Procedures	
		Raw Material Control	
		Equipment Calibration and Maintenance	
		Batch Records and Documentation	
		In-process Quality Control	
		Product Testing and Release	
		Stability Testing	
		Recall Procedures	
		Validation and Qualification	
Process Control	Temperature	Cleaning and Sanitization Environmental Conditions Quality Control Testing Macroscopic examinations Microscopic examinations	Davids and Bagley (2014); Cox et al. (2019); Huaulmé et al. (2020); Kjelkenes et al. (2022)
	Time pH		
	Pressure		
	Solvent-to-Solid Ratio		
	Mixing Intensity		
	Drying Conditions		
	Particle Size		
In-process Testing	Moisture Content	Chemical analysis Microbial testing Dissolution testing Physical measurements	Sridharan et al. (2016); Benedetti et al. (2019); Kjelkenes et al. (2022); Liang et al. (2023)
	Solvent Concentration pH Level		
	Mixing Time		
	Particle Size		
	Homogeneity		
	Pressure		
	Temperature		
	Weight Variation		
	Disintegration Time		
	Uniformity of Dosage Units		
	Residual Solvents		
Contamination Control	Microbial Contamination	Aseptic techniques	Dworkin et al. (2018); Noh et al. (2018); Ling et al. (2021); Prata et al. (2021); Jenner et al. (2022)
	Heavy Metals	Environmental Monitoring	
	Pesticide Residues	Equipment Cleaning and Sanitization	
	Mycotoxins	Packaging Material Evaluation	
	Solvent Residues	Good Manufacturing Practices	
	Foreign Matter	Quality Control Testing	
	Cross-Contamination		

#### 3.3.1 Raw material evaluation

The quality assessment starts with a thorough evaluation of the raw materials, which are the herbs used in the product (Liang et al., 2023). Raw material evaluation methods includes checking the identity, authenticity, purity, and quality of the herbs, which involve a combination of techniques and tests to assess the quality, authenticity, and safety of raw materials used in the production of herbal medication products (Sridharan et al., 2016; Benedetti et al., 2019). First of all, it is an organoleptic evaluation that involves the examination of raw materials using the senses, including sight, smell, taste, and touch (Sridharan et al., 2016). It helps identify any abnormalities or inconsistencies in appearance, odor, and taste. Next, a microscopic examination of plant material can help verify its identity and detect any adulteration or foreign substances (Saletnik et al., 2021; Zhang et al., 2021). High-performance thin-layer chromatography (HPTLC) is used to separate and identify different chemical components in a sample. It is often employed to verify the presence of specific markers or active compounds in herbal raw materials (Vestal, 1984; Hussain et al., 2015; Sontag et al., 2019). Fourier transform infrared spectroscopy (FTIR) is used to identify functional groups in raw materials, helping to assess their chemical composition (Agustika et al., 2022; Barnes et al., 2023). Atomic absorption spectroscopy (AAS) is employed to detect and quantify the presence of heavy metals in raw materials, ensuring they are within safe limits (Qu et al., 2016; Ortegón et al., 2022). High-performance liquid chromatography is utilized for quantitative analysis of active compounds in herbal materials, ensuring that they meet specified quality standards (Olsson et al., 2014; Xie et al., 2014). And total organic carbon (TOC) analysis is used to determine the total amount of organic carbon present in a sample, which can indicate the presence of contaminants (Shao et al., 2022; Lee et al., 2023). By using a combination of these raw material evaluation methods, manufacturers can ensure the quality, authenticity, and safety of the herbal ingredients used in their products. These evaluations are crucial for maintaining consistent product quality, meeting regulatory standards, and providing consumers with safe and effective herbal medication products.

#### 3.3.2 Good manufacturing practices for herbal products

Good Manufacturing Practices (GMP) guidelines are essential to the herbal industry to maintain product quality and protect consumer health (Lesch et al., 2021). GMP for herbal products is a set of guidelines and principles that ensure the quality, safety, and consistency of herbal medicines, supplements, and other herbal products (Castiglia et al., 2018). It involves raw material sourcing and identification, facility and equipment, standard operating procedures, batch records and documentation, quality control testing, validation and qualification, stability testing, recalls and complaints, regulatory compliance, and continuous improvement (Boyd, 1994; Jacquemart et al., 2016; Cundell et al., 2023). GMP also involve proper documentation, personnel training, hygiene practices, and quality control procedures to ensure consistent quality throughout the manufacturing process (Castiglia et al., 2018). GMP requires the use of high-quality, authentic, and properly identified herbal raw materials. Suppliers should be carefully selected and qualified to ensure the consistency and purity of the ingredients (Lesch et al., 2021). GMP-compliant facilities should be designed, maintained, and operated in a manner that prevents cross-contamination, ensures cleanliness, and provides a controlled environment for manufacturing (Melethil, 2006; Bai et al., 2022). GMP emphasizes the development and implementation of written standard operating procedures for all critical manufacturing processes (Shukla and Gottschalk, 2013). These procedures help ensure consistent and controlled production. Detailed batch records and documentation should be maintained for each product manufactured (Mager et al., 2007). This includes information about raw materials, manufacturing steps, quality control tests, and any deviations or corrective actions taken during production (Deagle et al., 2017; Smith, 2020). GMP requires routine quality control testing of raw materials, in-process samples, and finished products (Morgan et al., 2015). Testing may include the identification of herbal ingredients, quantitative analysis of active compounds, and evaluation of contaminants. Processes, equipment, and analytical methods used in herbal product manufacturing should be validated to demonstrate their effectiveness and accuracy (Williams et al., 2012). Herbal products should undergo stability testing to determine their shelf life and storage conditions (Cundell et al., 2023). This helps ensure that the product retains its quality and potency throughout its designated shelf life. Regular audits, self-inspections, and reviews of manufacturing processes help identify areas for enhancement and ensure ongoing compliance with GMP principles (Deagle et al., 2017). Finally, adhering to good manufacturing practices is essential for the herbal products industry to maintain product quality, safety, and consistency. GMP guidelines promote the use of standardized procedures, robust quality control, and proper documentation to ensure that herbal products meet the required standards and are safe for consumers (Smith, 2020). By following GMP principles, manufacturers can build trust with consumers, healthcare professionals, and regulatory authorities, contributing to the growth and acceptance of herbal products in the healthcare market.

#### 3.3.3 Process control

**FIGURE 3:**
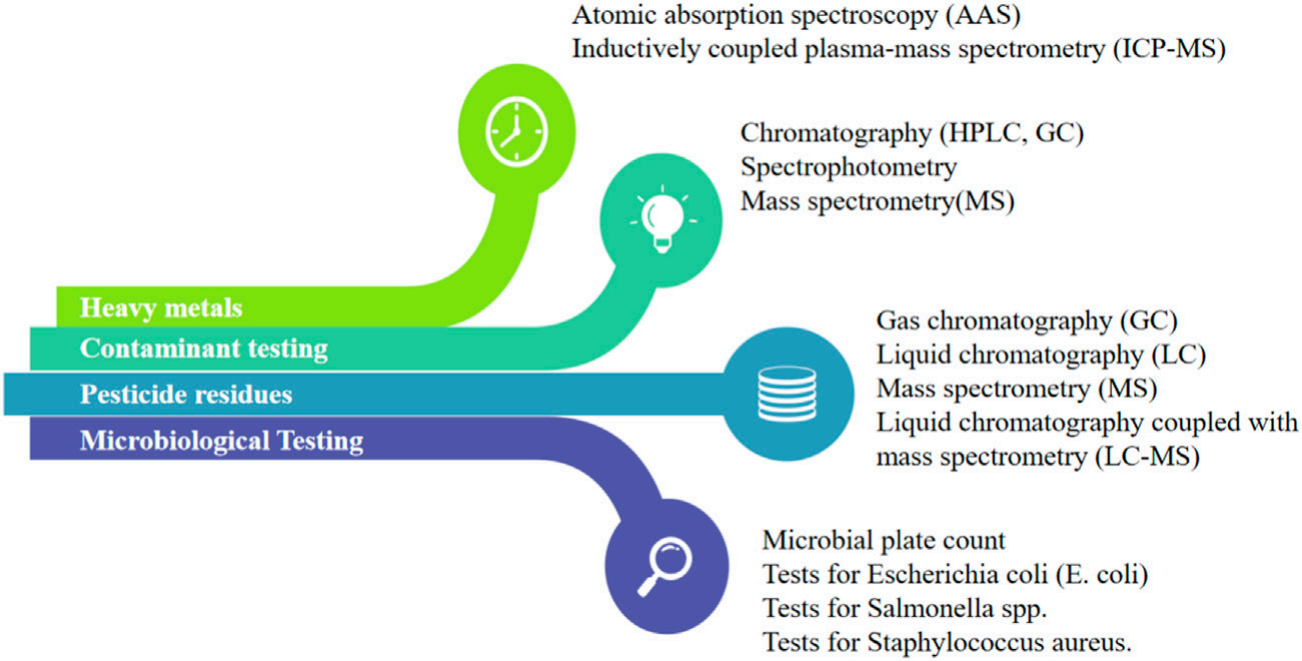
The quality control methods for contaminants, heavy metals, pesticide residues, and microbiology.

Monitoring and controlling critical parameters during processing is essential to maintain product quality (Qu et al., 2021). This includes parameters such as temperature, pressure, pH, mixing time, and drying conditions (Morgan et al., 2015). Regular monitoring and documentation of these parameters help identify any deviations from the desired specifications and allow for necessary adjustments or corrective actions to ensure product consistency (Wasalathanthri et al., 2020). Temperature control is vital in many processes as it directly impacts chemical reactions, phase changes, and microbial growth (Reischauer et al., 2009). Precise temperature control ensures that reactions proceed as intended, preventing unwanted by-products and ensuring the desired product quality. In pharmaceutical manufacturing, maintaining the correct temperature during drug synthesis helps produce stable and effective medications (Pan et al., 2019). Pressure control is particularly important in processes involving gases or liquids (Seo and Shin, 2022). Too much or too little pressure can affect reaction rates, solubility, and the overall efficiency of the process. In applications like chemical reactions, pressure control helps maintain a safe operating environment and prevents equipment failures (Schmidt et al., 2023). pH is a measure of acidity or alkalinity and significantly influences the stability and functionality of many products (Wright, 2021). In industries like food and beverage production, pharmaceuticals, and cosmetics, maintaining the correct pH level is critical for product preservation, taste, and effectiveness (Wiseman et al., 2022). For instance, certain enzymes are only active within specific pH ranges, making pH control essential during enzyme-based processes (Daniel et al., 2022). In processes involving mixing or blending, the duration and intensity of mixing time directly affect product homogeneity and consistency (Hernandez and Perera, 2022). Controlling the mixing time ensures uniform distribution of ingredients, which is crucial in formulations such as pharmaceutical tablets or food products (Adra et al., 2022). Drying is a common step in herbal medication industries, such as product processing, pharmaceuticals, and chemical manufacturing. Controlling drying conditions can change product defects, increase shelf life, or even keep fresh, which involves temperature, humidity, airflow, moisture content, texture, and stability (da Cunha et al., 2021). Quality testing for herbal medication products involves rigorous analysis to ensure the absence of contaminants, heavy metals, pesticide residues, and microbiological safety evaluation (Figure 3). By controlling critical parameters, manufacturers can achieve consistent product quality across different batches, leading to reliable and predictable outcomes, reducing waste and production time, avoiding product defects and minimizing rejections, and increasing its market value and consumer satisfaction.

#### 3.3.4 In-process testing

In-process testing involves performing quality tests at various stages of the manufacturing process to assess the product’s quality and consistency (Silva et al., 2022). These tests may include chemical analysis, microbial testing, dissolution testing, or physical measurements. In-process testing helps identify any variations or issues during processing, allowing for timely corrective actions and ensuring the final product meets the desired quality standards (Chan and Choo, 2013). The presence of contaminants, heavy metals, pesticide residues, and microbiological safety evaluation is a crucial major concern in herbal medication products (Omeje et al., 2021). Therefore, the detection of pollutants, pesticides, microbiological counts, and heavy metals in the environment is key to ensuring the quality of herbal medication products (Varol and Sünbül, 2017). Contaminant testing includes assessing the presence of impurities such as aflatoxins, mycotoxins, residual solvents, and environmental pollutants (Simpson and McKelvie, 2009). Various analytical techniques like chromatography (HPLC, GC), spectrophotometry, and mass spectrometry are employed to detect and quantify these contaminants (Levy et al., 2018). Heavy metals such as lead, mercury, arsenic, and cadmium can pose health risks if present in herbal medication products. Testing methods such as atomic absorption spectroscopy or inductively coupled plasma-mass spectrometry (ICP-MS) are utilized to measure heavy metal content and ensure compliance with permissible limits (Lockwood et al., 2021). Pesticide residues can result from agricultural practices or contamination during cultivation. Analytical techniques like gas chromatography (GC) or liquid chromatography (LC) coupled with mass spectrometry (MS) are employed for pesticide residue analysis, ensuring adherence to regulatory standards (Lockwood et al., 2021). Microbiological testing assesses the presence of harmful microorganisms in herbal medication products (Levy et al., 2018). It includes testing for total microbial counts, specific pathogens, and the absence of certain indicator organisms (Levy et al., 2018). Common methods include microbial plate count, tests for *E. coli* (*Escherichia coli*), *Salmonella* spp., and *Staphylococcus aureus* (York et al., 2000). The microbial evaluation for total coliform counts (TCC), total viable counts (TVC), and total yeast and mold counts (TYMC) was evaluated using the method described in the compendium of methods for microbiological testing of herb medication products described by Hervert and Alles (2016) with minor modifications. These tests ensure product safety and compliance with microbial limits set by regulatory authorities.

Analytical techniques are employed to determine the presence, identity, and concentration of active constituents in herbal medication products (Seo and Shin, 2022). High-Performance liquid chromatography is widely employed to separate, identify, and quantify individual chemical components, including active compounds, in herbal extracts (Wright, 2021). It provides precise measurements and is useful for standardization and quality control. Gas chromatography-mass spectrometry is used for volatile compounds and essential oils analysis in herbal medication products (Silva et al., 2022). It enables the identification and quantification of specific compounds based on their mass spectra and retention times. Thin-Layer Chromatography is a rapid and cost-effective technique used for qualitative analysis and identification of compounds in herbal medicines (Del Bubba et al., 2013). It involves separating components based on their differential migration on a thin layer of adsorbent material. Spectroscopic Techniques like UV-Vis spectroscopy, infrared (IR) spectroscopy, and nuclear magnetic resonance (NMR) spectroscopy are employed for qualitative and quantitative analysis of herbal constituents. They provide information on chemical structures, functional groups, and concentrations. These quality testing and analytical techniques help ensure the safety, efficacy, and consistency of herbal medication products. By performing these tests, manufacturers can identify potential contaminants, verify active constituent content, and ensure compliance with regulatory requirements, thereby ensuring the quality and safety of the products.

#### 3.3.5 Contamination control

Contamination control is a critical aspect of various herbal medication industries, ensuring safety, product quality, and environmental protection (Raeisossadati et al., 2016). Contamination control methods are techniques and practices employed to prevent, minimize, or eliminate the presence of harmful or unwanted substances in a specific environment, product, or process (Edberg et al., 1989; Simpson and McKelvie, 2009). These methods vary depending on the industry and the nature of the contamination. This includes implementing measures such as cleaning and sanitation, sterilization, disinfection, hand hygiene, cleanroom technology, air filtration, regulatory compliance, and environmental monitoring (Wan et al., 2019). Regular cleaning and sanitation of surfaces, equipment, and facilities are fundamental contamination control methods. Proper cleaning removes dirt, debris, and potential contaminants, reducing the risk of cross-contamination. Sterilization is a critical method used in healthcare settings and the pharmaceutical industry to eliminate all viable microorganisms, including bacteria, viruses, and spores. Techniques like autoclaving, irradiation, and chemical sterilization are commonly used. Disinfection involves the use of chemical agents to reduce the number of microorganisms on surfaces and objects. It is commonly used in healthcare settings and food production to control the spread of pathogens. Clean-rooms are controlled environments designed to minimize airborne particulates and microorganisms (Safra, 2019). They are widely used in industries like semiconductor manufacturing, aerospace, and pharmaceuticals. High-efficiency particulate air (HEPA) filters are used in ventilation systems to remove airborne particles, including microorganisms and other contaminants. In industries like food production and healthcare, pest control measures are essential to prevent contamination from insects and rodents. Many industries have specific regulations and standards related to contamination control (Panteghini et al., 2001). Compliance with these regulations is essential to ensure public safety, product quality, and environmental protection. Regular monitoring of environmental conditions, such as temperature, humidity, and air quality, can help identify potential sources of contamination and ensure appropriate control measures are in place (Omeje et al., 2021). Contamination control methods encompass a range of practices and techniques aimed at preventing or reducing contamination in herbal medication products industries (Levy et al., 2018). These methods are critical to ensure the safety, quality, and efficacy of products, protect public health, and preserve the environment.

## 4 Discussion

**FIGURE 4:**
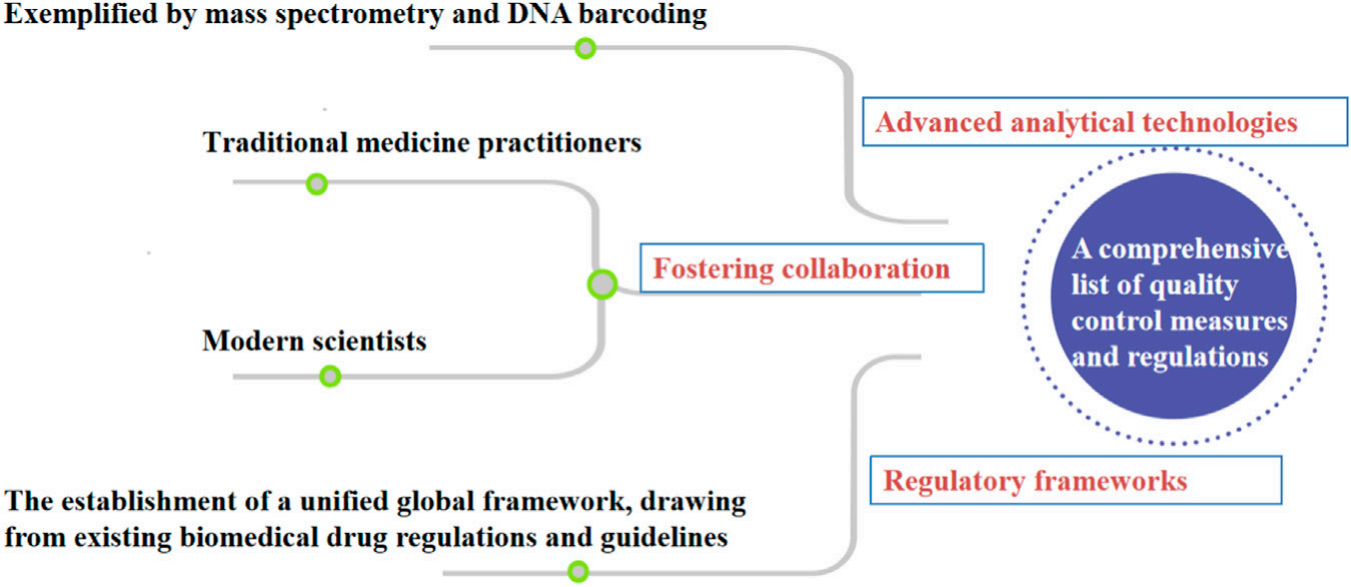
A diagram of the road map for the comprehensive list of quality control measures and regulations applied to herbal medicines.

Despite the growing recognition of herbal medicines’ therapeutic potential, the field of quality control in this domain faces several critical gaps and challenges that warrant diligent attention. One notable challenge is the significant variability in product composition and quality due to factors such as geographical variations, cultivation methods, and post-harvest processing. This inherent diversity poses a unique hurdle for establishing consistent quality standards across different herbal products. Rapid advancements in analytical technologies, such as mass spectrometry and DNA barcoding, have transformed the landscape of quality control in other industries, yet their integration into herbal medicine quality assessment has been sporadic and inconsistent. We propose the utilization of molecular fingerprinting techniques, such as DNA bar-coding and metabolic, to authenticate herbal ingredients. These approaches offer accurate species identification and enable the detection of adulterants or contaminants. Secondly, we advocate for the incorporation of real-time monitoring and Internet of Things (IoT) devices along the herbal supply chain. This enables continuous data collection on environmental conditions, ensuring the preservation of botanical integrity and product quality. Besides, Data-driven predictive modeling is also necessary for quality control practices in the herbal industry. Introducing data-driven predictive models, including machine learning and artificial intelligence, can forecast the impact of processing variables on herbal product quality. This empowers manufacturers to optimize production processes for consistent outcomes. Finally, the collaboration between regulatory bodies, herbal practitioners, and manufacturers is proposed to establish comprehensive industry standards. These standards would encompass cultivation, harvesting, processing, and quality control, ensuring adherence to best practices. Moreover, the under-utilization of traditional knowledge is a missed opportunity for elevating the quality control process. Traditional healers and local communities possess invaluable insights into the therapeutic properties and usage of herbs. Integrating this wisdom with modern scientific approaches could enrich the quality control paradigm and enhance product efficacy. In response to these challenges, a holistic approach to quality control is needed—one that bridges the gaps between traditional practices and modern innovations, streamlines regulatory oversight, and nurtures collaborative partnerships among stakeholders. To illustrate the interplay of these elements, a diagram of the road map is presented below in Figure 4. By following this roadmap, herbal medicine manufacturers and regulators can strengthen the safety and efficacy of herbal medication products, thereby instilling confidence in consumers and fostering the responsible growth of the herbal medicine industry. The road map also serves as a foundation for continuous research, improvement, and knowledge-sharing, ultimately contributing to the advancement of quality control practices for herbal medicines. medicines.

Herbal medicines often draw from centuries of traditional knowledge, which should be thoughtfully integrated into modern quality control practices. It is worth noting that some of the quality control advances suggested for herbal medicines are already well-established practices in the context of biomedical drug products. Building upon the insights highlighted by previous researchers, strategic integration of cutting-edge analytical techniques, harmonized global standards, and collaborative partnerships emerges as the pathway forward. While some aspects of biomedical drug quality control can serve as a model for herbal medicines, there are specific differences between the two domains. Although adopting certain aspects of biomedical drug quality control can be beneficial for the quality control of herbal medicines, the application of such measures to herbal medicines raises distinct considerations. By juxtaposing the existing practices with the proposed enhancements, we underscore the potential evolution of quality control measures for herbs (Table 3). By embracing these enhancements, we hold the promise of transforming the herbal medicine landscape and instilling newfound confidence in consumers seeking natural remedies.

**TABLE 3:** A potential evolution of quality control measures.

Quality control aspect	Current practices	Biomedical drug products	Herbal medicines	Enhanced approach for herbal medicines
Analytical Testing Methods	Visual inspection, basic impurity tests	High-precision tests, such as HPLC and GC-MS, to identify and quantify active compounds	Traditional visual inspection and basic tests for impurities and contaminants	Adoption of advanced technologies like mass spectrometry and DNA barcoding to authenticate herbal components
Regulatory Framework	Varied regional regulations	Stringent global regulations and guidelines (e.g., FDA, EMA) for drug approval and manufacturing	Varied regulations across regions, with potential gaps in standardization	Establishment of a unified global framework based on international guidelines and good manufacturing practices
Collaboration & Knowledge	Limited collaboration between stakeholders	Collaboration between scientists, researchers, and pharmaceutical companies	Limited integration of traditional knowledge and modern research	Encouragement of multidisciplinary partnerships between traditional practitioners, scientists, and regulators, harnessing both traditional knowledge and modern expertise

**FIGURE 5:**
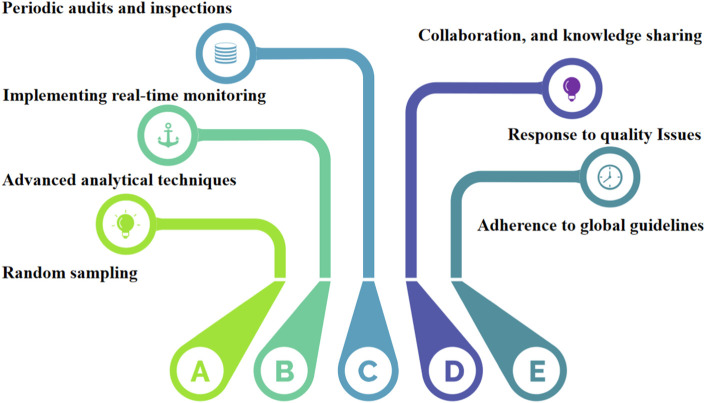
A map for the quality of herbal medicine products control of enterprises.

By synthesizing a comprehensive understanding of the challenges and opportunities within the realm of herbal medicine quality control, we equip enterprises with actionable insights to enhance their practices such as a comprehensive review of quality control measures, identification of gaps and challenges, recommendations for enhancements, integration of modern techniques, regulatory harmonization, collaborative approach, continuous improvement, and innovation (Figure 5). Enterprises learn how to maintain uniformity in active ingredients, dosage forms, and potency and improve the core aspects of GMP, including cleanliness, personnel training, documentation, and equipment maintenance. This knowledge empowers enterprises to conduct rigorous quality assessments before products reach the market. By underscoring the role of quality control in ensuring safety, the text assists enterprises in identifying potential risks associated with herbal medicine production. Enterprises are encouraged to regularly review and update their quality control processes in light of emerging research, technological advancements, and changing consumer preferences. By implementing the insights provided in the text, enterprises can confidently navigate the complexities of herbal medicine production and contribute to the availability of safe and consistent products for consumers.

## 5 Conclusion

Quality control for the efficiency and safety of herbal medication products is of significant importance to safeguard consumer health, establish reliable treatment options, comply with regulations, and foster the growth of the herbal medicine industry. The efficacy of herbal medications depends on the presence and concentration of active compounds. Quality control processes should focus on establishing standardized procedures for sourcing, processing, and formulating herbal ingredients. Besides, regular monitoring of raw materials and finished products, quantitative analysis of these compounds to guarantee consistent therapeutic effects, rigorous testing for potential contaminants, implementing GMP principles in the production process, and post-market surveillance are often attributed to ensure safety and efficacy. With continuous quality control measures in place, any issues related to product quality or safety can be identified and addressed promptly. Advanced techniques such as DNA barcoding can be employed to verify the authenticity of herbs used in the products. This proactive approach minimizes the risk of widespread product recalls or adverse events.

Ensuring the quality, efficiency, and safety of herbal medication products involves standardization, authentication, contaminant testing, quantification of active compounds, adherence to regulations, stability testing, GMP implementation, validated methods, post-market surveillance, and open collaboration among stakeholders. These key findings and takeaways provide a road map for establishing robust quality control processes for herbal medication products.

In conclusion, this comprehensive review underscores the pivotal role of quality control in herbal medication products, emphasizing its significance in ensuring efficiency and safety. The use of robust quality control methods ensures the authenticity and therapeutic value of herbal remedies, fostering consumer trust and promoting the responsible integration of herbal medicine into modern healthcare practices. Continued research and collaboration between traditional knowledge and modern science will undoubtedly enhance the quality and acceptance of herbal medication products, further benefiting public health and wellbeing.

## Abbreviations

AAS, Atomic absorption spectroscopy; *E. coli*, *Escherichia coli*; FTIR, Fourier transform infrared spectroscopy; GC, Gas chromatography; GC-MS, Gas Chromatography-Mass Spectrometry; GMP, Good manufacturing practices; HEPA, High-efficiency particulate air; HPLC, High-performance liquid chromatography; HPTLC, High-performance thin-layer chromatography; ICP-MS, Inductively coupled plasma-mass spectrometry; IRS, Infrared spectroscopy; IoT, Internet of things; LC, Liquid chromatography; MS, Mass spectrometry; NMR, Nuclear magnetic resonance; NMRS, Nuclear magnetic resonance spectroscopy; TCM, Traditional chinese medicine; TLC, Thin-Layer Chromatography; TCC, Total coliform counts; TOC, Total organic carbon; TVC, Total viable counts; TYMC, Total yeast and mould counts; UVVS, UV-Vis spectroscopy.
